# Heart Rate and Heart Rate Variability in Dairy Cows with Different Temperament and Behavioural Reactivity to Humans

**DOI:** 10.1371/journal.pone.0136294

**Published:** 2015-08-20

**Authors:** Levente Kovács, Fruzsina Luca Kézér, János Tőzsér, Ottó Szenci, Péter Póti, Ferenc Pajor

**Affiliations:** 1 MTA–SZIE Large Animal Clinical Research Group, Üllő-Dóra major, Hungary; 2 Institute of Animal Husbandry, Faculty of Agricultural and Environmental Science, Szent István University, Gödöllő, Hungary; 3 Department and Clinics of Reproduction, Faculty of Veterinary Science, Szent István University, Budapest, Hungary; University of Parma, ITALY

## Abstract

From the 1990s, extensive research was started on the physiological aspects of individual traits in animals. Previous research has established two extreme (proactive and reactive) coping styles in several animal species, but the means of reactivity with the autonomic nervous system (ANS) activity has not yet been investigated in cattle. The aim of this study was the characterization of cardiac autonomic activity under different conditions in cows with different individual characteristics. For this purpose, we investigated heart rate and ANS-related heart rate variability (HRV) parameters of dairy cows (N = 282) on smaller- and larger-scale farms grouped by (1) temperament and (2) behavioural reactivity to humans (BRH). Animals with high BRH scores were defined as impulsive, while animals with low BRH scores were defined as reserved. Cardiac parameters were calculated for undisturbed lying (baseline) and for milking bouts, the latter with the presence of an unfamiliar person (stressful situation). Sympathetic tone was higher, while vagal activity was lower in temperamental cows than in calm animals during rest both on smaller- and larger-scale farms. During milking, HRV parameters were indicative of a higher sympathetic and a lower vagal activity of temperamental cows as compared to calm ones in farms of both sizes. Basal heart rate did not differ between BRH groups either on smaller- or larger-scale farms. Differences between basal ANS activity of impulsive and reserved cows reflected a higher resting vagal and lower sympathetic activity of reserved animals compared to impulsive ones both on smaller- and larger-scale farms. There was no difference either in heart rate or in HRV parameters between groups during milking neither in smaller- nor in larger-scale farms. These two groupings allowed to draw possible parallels between personality and cardiac autonomic activity during both rest and milking in dairy cows. Heart rate and HRV seem to be useful for characterisation of physiological differences related to temperament and BRH.

## Introduction

Knowledge of the behavioural characteristics of dairy cows is important for the breeding, housing and management of animals. Behavioural and physiological differences between individuals in response to a stressor or an environmental challenge are often described with the terms ‘coping style’ [1,2] and ‘temperament’ [3,4].

The term ‘temperament’ is frequently used to describe the relatively stable differences in the behavioural predisposition of animals, which can be related to psychobiological mechanisms [5,6]. According to Burrow [7], temperament is simply an animal’s behavioural response to handling by humans, while others defined this term as an animal’s main personality or mood trait in relation to humans [8]. For coping, several definitions are given. Earlier reports referred to coping as the behavioural and physiological efforts to master the situation [9,10]. Farm animals kept in intensive housing systems use a set of strategies (escape, remove, search, wait) to cope with aversive situations [1]. From the point of view of stress research, generally two response patterns may be distinguished in animals with different coping styles [11]. The first type is the active response, which was originally described by Cannon [12] as the ‘fight or flight’ response. Behaviourally, this active response is characterized by territorial control and aggression. Engel and Schmale [13] described the second type of stress response as the conservation-withdrawal response, which is characterized behaviourally by immobility and low levels of aggression. Based on a review on coping styles in animals [10], it is preferable to use the terms *proactive coping* rather than active coping and *reactive* rather than passive coping.

Since the 1980s, an increasing number of studies have focused on individual differences related to different coping strategies or on differences in temperamental traits in several species in response to challenges using behavioural tests [14,15]. Extensive research has been done on farm animals using human exposure as a stressor to evaluate temperament and behavioural reactivity, which are mainly based on evaluation of the animal’s personal area [16]. The tests used for cattle were originally designed for laboratory animals, generally without taking the biological significance of dairy cows into account.

Besides behavioural reactions, parameters of heart rate variability (HRV), i.e., the short-term fluctuations in the variability of successive cardiac interbeat intervals (IBI) were also found useful to differentiate between individual traits in rats [17], poultry [18], horses [19], and cattle [4]. However, little is known about behavioural traits of dairy cattle and associated basal physiology for the description of individuals. It is therefore important to understand how cattle perceive their environment and how this is related to physiological markers such as HRV. While temperament is a relatively simple individual trait in domestic animals [14], coping is regarded as a multidimensional characteristic, including a series of components such as flexibility [20], immobility or adaptation [21]. Although studies on coping styles emphasize the multidimensional character of individual traits, reactivity is often found to be an important dimension [22]. Besides several components, in dairy herds, one important dimension may be the animal’s reactivity to people. In this paper, we prefer to use the term behavioural reactivity to humans (BRH).

Based on the landmark article of Koolhaas et al. [10] according to which animals with different behavioural reactivity are differing in autonomic nervous system (ANS) activity our main goal was the characterization of cardiac autonomic activity under different conditions in cows that differed in temperament and BRH. For this purpose, we calculated ANS-related HRV indices under different conditions: 1) while animals were lying undisturbed (baseline) and 2) during milking with the presence of an unfamiliar person (stressful situation). As low parasympathetic activity (and high sympathetic activity) was found to be associated with high emotional reactivity in horses [23], and it seems that HRV and temperament are associated [24, 25] we hypothesized higher sympathetic and lower vagal activity in temperamental cows than in calm ones during both rest and milking. We also presumed that cows showing withdrawal behaviour (a major component of reactive coping style [13]) during the BRH-test would be characterized by higher basal vagal and lower sympathetic activity (as it was found in rats by Sgoifo et al. [17]) than animals that show no attempt to increase the distance from humans and react more active.

## Materials and Methods

### Animals and Housing

This study was approved by the Department of Epidemiology and Animal Protection of the Directorate of Food Chain Safety and Animal Health at the Central Agricultural Office (permission no. 22.1/1266/3/2010). All procedures involving animals were specifically approved by the Ethics Committee of the Faculty of Veterinary Science, Szent István University.

A total of 282 multiparous lactating Holstein-Friesian cows were included in this study. Cows were selected randomly from six Hungarian farms. Farm ‘A’ (Hatvan-Kerekharaszt, József major: 47°66'30.2"N, 19°62'42.6"E), farm ‘B’ (Nóráp, family farm: 47°16'23.5"N, 17°27'28.7"E) and farm ‘C’ (Ráckeresztúr, Lászlópuszta, 47°26'57.5"N, 18°83'58.6"E) had a herd comprising 70–80 lactating cows (hereinafter called *smaller-scale farms*), farm ‘D’ (Etyek, Ödön major, 47°42'78.4"N, 18°77'39.9"E) and farm ‘E’ (Jászapáti, 47°53'96.9"N, 20.14'53.9"E) had around 1500 cows, while farm ‘F’ (Beremend, Csípőtelek, 45°48'54.2"N, 18°28'23.4"E) had a herd of 1700 animals (hereinafter called *larger-scale farms*). Experimental animals (farm ‘A’: N = 43; farm ‘B’: N = 45; farm ‘C’: N = 46; farm ‘D’: N = 48; farm ‘E’: N = 48; farm ‘F’: N = 52) were balanced for age, stage of pregnancy, number of lactations, days in milk and body condition score both for smaller-scale and larger-scale herds. Average daily milk yield was 27.40 ± 2.35 kg for cows from smaller-scale farms and 34.25 ± 3.10 kg for cows from larger-sized farms. All animals included in the study were clinically healthy. Cows in oestrus were excluded from the study.

All cows were reared under loose housing conditions in freestall barns. Group size, space allowance per animal, bedding and the applied milking system were similar for several smaller- and larger-scale farms. Animals were fed twice a day on each farm (08:00–09:00 a.m. and 17:00–18:00 p.m.) and had free access to water. Cows were milked twice daily on each farm, with the exception of farm ‘E’ where cows were milked around 13:00 p.m. as well. The cows were habituated to the applied milking system on each farm. On smaller-scale farms 2×5 stall herringbone milking parlours were in operation, while larger-scale farms worked with 2×2×22-stall parallel parlours. Human contact was limited to necessary management routines before and during the experiment on each farm.

### Measurement Preparation

The experiment was carried out during two trials: (1) between September and November 2012 for smaller-scale farms, and (2) between September and November 2013 for large-scale farms. Each farm was visited for a 3-week period. Data were collected from each animal on three consecutive days, and eight cows were involved in each trial for 24-hour IBI recordings.

IBIs were recorded continuously with the Polar Equine RS800 CX mobile recording system (Polar Electro Oy, Kempele, Finland), with two integrated electrodes and a specific transmitter. Devices were fitted to cows on each farm in individual insemination stalls after the morning milking. After soaking the body surface under the electrodes with tap water, electrode sites were covered with ultrasound transmission gel (Aquaultra Blue, MedGel Medical, Barcelona, Spain). The transmitters and the two electrodes were then positioned as advised in an earlier review [26]. Preparations were done in the morning, approximately between 07:00 and 08:00 a.m., after milking. IBI recordings started two hours after preparations to allow the animals time to get adapted to wearing the devices. After moving cows to the lying cubicles, any kind of disturbance or any unnecessary contact with animals throughout the data collection period was avoided.

### Behavioural sampling

Posture of the cows was video recorded throughout the study on each farm with a closed-circuit camera system including two day/night outdoor network bullet cameras (Vivotek IP8331, VIVOTEK Inc., Taiwan). The cameras were installed above the experimental areas in a way that gave the best possible view of the animals to help match the start and the end points of the 5-min samples for later HRV analysis (see the section ‘Processing of IBI data’ for details).

The behaviour displayed by the animals during the behaviour tests was video recorded with two portable digital video cameras (Legria HF M36, Canon, Japan). Two tests were performed during the experiment to evaluate the animals’ (1) temperament (when cows were restrained) and (2) behavioural reactivity to humans (BRH, without tethering). Temperament was roughly scored directly by three independent judges while fixing the heart rate devices, and categorized (Table 1). Because none of the cows was exposed to a similar experience before, we thought that this procedure could serve as an appropriate test for the assessment of temperament. Following the procedure, a mean score for each animal was calculated.

**Table 1:** Restlessness behaviour scores based on the temperament test.

Description of behaviour	Score	Temperament categories
Standing calm, only ear hanging (duration in seconds), tail flicking and/or infrequent muscle contractions are observed	1	Calm
Frequent ear hanging and muscle contractions, stretching the neck backwards	2	Intermediate
Head shaking and/or defence reactions (kicking, butting) or fall (the cow collapses to the ground onto both carpi)	3	Temperamental

BRH was scored in the familiar environment of the animals (while cows were standing or eating at the feeding bunk) as recommended in the review of Forkman et al. [15], since stimuli of an unfamiliar testing environment can affect behavioural reactions. Our goal was to generate a new test through modifying an approach test [27] by keeping its strengths but avoiding as many of the weaknesses of open field tests as possible. In this test, we integrated both the intensity and the quality of the behavioural response. The test was done by an unfamiliar person who wore the same clothing during the testing. The experimenter held her arm overhand, looking at the muzzle, and waited for attention of the focus cow. Then the experimenter approached the cow slowly with constant speed (approx. 1 step/s, practiced before the experiment) from the front until the animal withdrew or until the cow was touched. The distance between the experimenter’s hand and the muzzle at the moment of withdrawal was determined based on video recordings (5 cm resolution). Withdrawal was categorized into a higher [stepping back (minimum two steps)] and a lower response [turning the head away (>90°)] (Table 2). Each animal was tested twice at an interval of at least 30 min. An average score of each cow was used for statistical analysis.

**Table 2:** Components of behavioural reactivity to humans (BRH) measured in this study.

Parameters of the tests	Behavioural reactions and scores			
*Avoidance distance (AD)*	0 cm: 1 score	5–40 cm: 2 score	>40 cm: 3 score	
*Reaction (R)*	stay: 1 score	turning the head away: 2 score	stepping back: 3 score	escape: 4 score
*Interaction (I)*	0 score: no interaction observed	1 score: touchable, smelling or stabbing the hand, animal can be stroked or fed from the hand		

Behavioural reactivity to the approaching person was evaluated as follows:

BRH=AD×0.6+R×0.4−I,

Based on the BRH scores three groups were formed:

0 < BRH ≤ 1: impulsive; 1 < BRH < 3: intermediate; 3 ≤ BRH: reserved

In our study, impulsive animals had to be touchable, since if the opposite of this had been true (AD ≥ 2), no interaction could have occurred (I = 0), and, therefore, the BRH score would be ≥ 2 × 0.6 = 1.2. Impulsive animals stood following contact, therefore in their case R = 1 (all focal animals showed either aggressive or positive reactions, in most cases positive). If R > 1 (the animal turned her head away or stepped back or even escaped), interaction also did not exist with the approaching person (I = 0), therefore the minimum BRH score was 1 × 0.6 + 2 × 0.4 = 1.4. A reserved animal’s AD was minimum 40 cm and they stepped back or escaped during tests without any interaction with the approaching person (minimum BRH score: 3 × 0.6 + 3 × 0.4 = 3). During the experiment, no escape behaviours were observed. The number of animals on each farm was balanced for both temperament and BRH groups.

During the evening milking another person, who was unknown to the cows, was present in the milking parlour. When the milker started to prepare the udder, the experimenter walked toward the cow until he stood directly in front of the cow, in contact with the front of the milking stall. The experimenter stood in this position until milking was finished (for details see the section ‘Processing of IBI data’) with his hands in his pockets, not looking at or touching the cow. Once the milking was completed, the experimenter left the stall.

### Processing of IBI data

**Fig 1:**
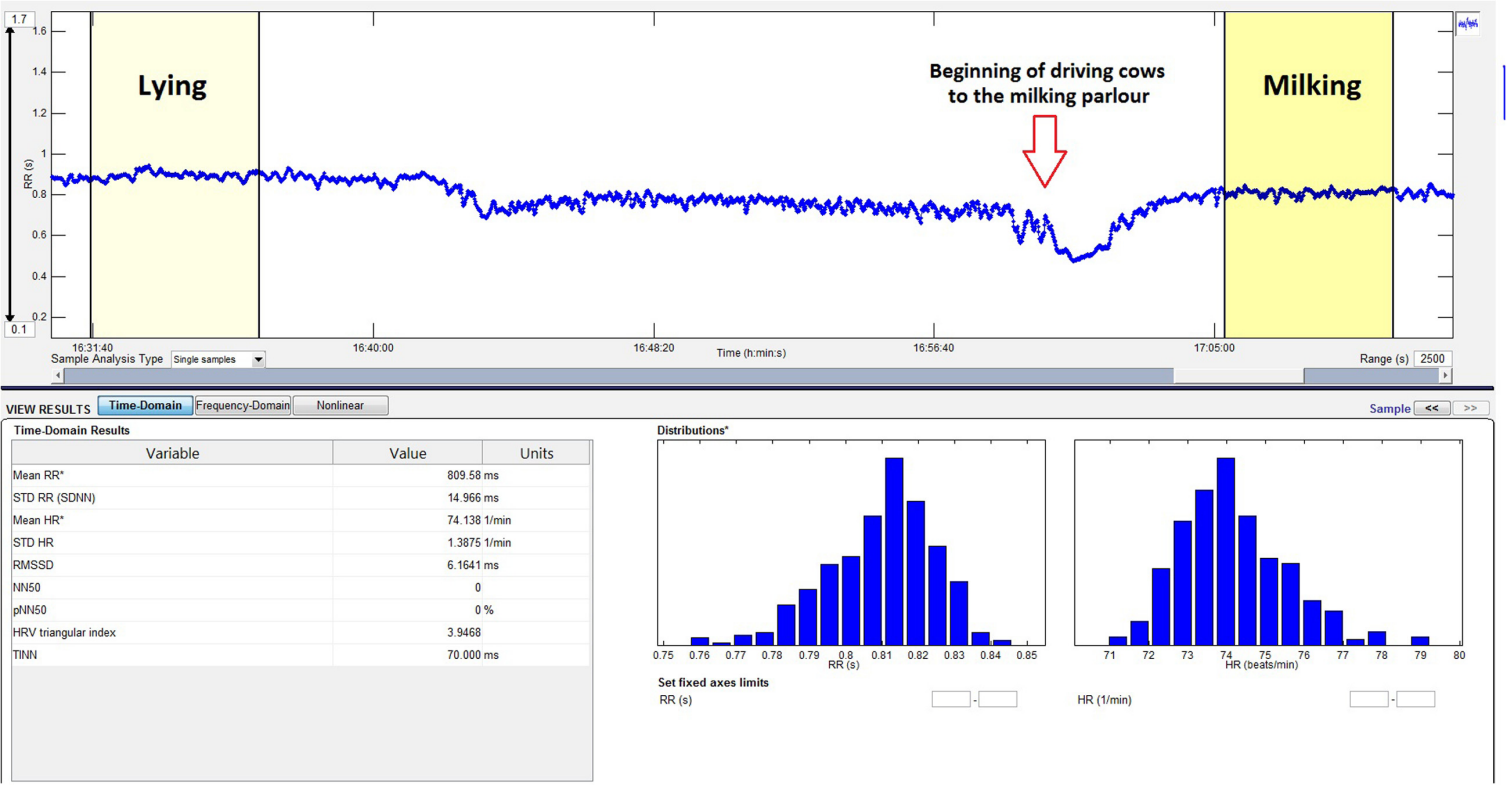
Selected lying and milking bouts from the IBI curve of an experimental animal. Time domain parameters (left) and the distribution of the length of IBIs and heart rate (right) calculated for the period of milking using the Kubios 2.2 HRV analysis software.

We calculated heart rate and HRV parameters from those IBI segments which were recorded while the animals were lying and being milked (Fig 1). The criteria of ‘lying idle’ were as follow: (1) the cow is lying comfortably in the cubicle without any disturbance from her herd mates; (2) the cow finished feeding and walking until 10 min before the start of data recording; (3) during the last 2 min before data recording and throughout the recording no environmental effects or disturbances were observed. We excluded from the analysis the data obtained 60 min after the animals were tethered for fixing the heart rate devices. During the 3-day sampling periods 12.37 ± 2.30 (mean ± SD, range 10–14, N = 134 cows) samples per cow were analyzed for smaller-scale farms and 13.72 ± 3.15 (mean ± SD, range 11–14, N = 148 cows) samples for larger-scale farms. IBI samples used for the calculation of baseline HRV parameters represented cardiac activity throughout the day on each farm, including the dark period. Samples chosen for analysis were balanced for three observation periods (09:00 a.m. to 16:00 p.m., 19:00 to 23:00 p.m., and 02:00 to 05:00 a.m.) for each animal on every investigated farm, since the time of the day influences cardiac activity [28].

IBI data recorded between the beginning of udder preparation (the first contact between the milker’s hand and the udder) and the removal of the last teat cup were chosen for the analysis of cardiac activity during milking. For this purpose, data collected during the evening milking were used. During the 3-day sampling period for a focal animal, 2.73 ± 0.22 (mean ± SD, range 2–3, N = 134 cows) samples per cow fulfilled the criteria of HRV analysis on smaller-scale farms and 2.60 ± 0.41 (mean ± SD, range 1–3, N = 148 cows) samples on larger-scale farms.

The HRV analysis was performed with the help of Kubios 2.2 HRV Software [29]. For artefact correction, the custom filter was used and set at 0.3, identifying IBIs differing from the previous IBI by more than 30% as artefacts. After abnormal interval removal, the algorithm of the program substitutes detected errors with interpolated intervals calculated from the differences between previous and next accepted IBIs. In addition, a visual inspection of the corrected data was performed to edit out any artefacts still existing (generally Type 4 and Type 5 errors, for details see Ref. [30]). For removing slow nonstationary trend components, the ‘smoothness priors’ based detrending approach was chosen with λ = 1000 and fc = 0.029 Hz. Time domain measures included mean heart rate and the vagus-related root mean square of successive differences (RMSSD) between the consecutive IBIs. As frequency domain parameters, the high-frequency component (HF) of HRV (presented in normalized units) and the ratio of the low-frequency (LF) and the HF components (LF/HF) were calculated by fast Fourier transformation (FFT). HF is a good marker of parasympathetic activity [31], whereas LF/HF provides essential information on the state of sympathovagal balance in farm animals [26]. Both parameters are frequently used in dairy cattle [32]. According to von Borell et al. [23], limits of the spectral components were set to 0.05–0.20 Hz for LF and to 0.20–0.58 Hz for HF. This frequency band width for the HF power was also used by earlier reports on HRV analysis in dairy cattle [33–36]. All parameters were calculated in equal time windows of 5 min for both undisturbed lying bouts and milking as advised by earlier reviews on HRV analysis using the FFT method [26,37].

### Statistical evaluation

Statistical analysis was performed using the SPSS 18 (SPSS Inc., Chicago, IL) statistical software. The analysis included two steps. At first, a General Linear Model univariate was used for detecting factors having possible effects on baseline heart rate and HRV values. To avoid pseudo-replication the averaged values of single samples collected for individuals were used for analysis. The model included cardiac parameters as dependent variables, while the number of lactation, days in milk and herd size (smaller-scale farms, larger-scale farms) were fixed factors. The model involved age of cows and milk yield on experimental days as covariate, while farms and experimental days as random factors. Before analysis, the Levene’s test was used for testing the equality of error variances. Tests of between-subjects effects were also determined for all sources of variances. The evaluation of main effects was adjusted by the Bonferroni *post hoc* test. The level of significance was set at 0.05. As heart rate and HRV indices were affected only by herd size (heart rate: *F =* 8.2, *P* = 0.020; RMSSD: *F =* 12.4, *P* = 0.006; HF: *F =* 56.8, *P* = 0.002; LF/HF: *F =* 56.8, *P* = 0.002; SD1: *F =* 6.9, *P* = 0.035), we compared cardiac parameters measured during lying and milking for temperament and BRH groups separately on smaller-scale and larger-scale farms.

The Shapiro-Wilk test for equality of variances was performed before the next step of analysis to check homogeneity of HRV parameters separately for smaller- and larger-scale farms. Baseline HRV values and HRV recorded during milking were then compared between temperament groups and BRH groups, respectively. The averaged values of single samples collected for each animal were used for analysis. In case of normal distribution of data, the Repeated Measures ANOVA was used and statistical significances between groups were calculated with Tukey’s *post-hoc* test (*P* < 0.05). In case of non-normality of data, differences between the various groups were tested by Friedman rank sum test with the Neményi *post-hoc* test (*P* < 0.05) for both smaller- and larger-scale farms.

## Results

### Temperament and cardiac autonomic activity

Heart rate and LF/HF were higher, while RMSSD and HF were lower in temperamental cows than in both calm and intermediate animals when measured during lying posture (Table 3). These differences were similar both on smaller- and larger-scale farms, reflecting the higher basal sympathetic and lower vagal activity of temperamental cows as compared to calm or intermediate ones. Differences were the most pronounced (*P* < 0.01) in spectral indices of HRV (HF and LF/HF). Slight differences were found between calm and intermediate cows in basal ANS activity mirrored only by HF (*P* < 0.05, on farms of both sizes) and LF/HF (*P* < 0.05, only on larger-scale farms). During milking, spectral parameters indicated a higher sympathetic and lower vagal activity of temperamental cows as compared to calm ones in farms of both sizes (higher LF/HF and lower HF, respectively). Heart rate was higher in temperamental cows than in calm ones during milking, but the difference was significant only on larger-scale farms. We found no differences between groups in RMSSD during milking (*P* > 0.05, regarding all comparisons).

**Table 3:** HRV parameters of dairy cows with different temperament calculated for lying and milking.

Temperament		HRV parameter								
		Heart rate (min^–1^)		RMSSD (ms)		HF (n.u.)		LF/HF	
*Smaller-scale farms*	N	Lying	Milking	Lying	Milking	Lying	Milking	Lying	Milking
Calm	43	63.5±2.6^a^	72.4±3.2	35.9±10.2^a^	19.3±6.8	62.4±8.6A^a^	28.4±6.5^a^	0.6±0.3^A^	2.3±0.3^a^
Intermediate	48	65.9±3.0^a^	75.6±3.8	33.2±7.4^a^	14.8±5.4	49.6±6.6^Ab^	23.7±5.6^ab^	1.0±0.5^Aa^	2.7±0.3^a^
Temperamental	43	73.1±4.1^b^	79.3±4.0	22.2±5.6^b^	15.4±4.4	32.1±4.1^B^	14.5±4.9^b^	2.1±0.8^Bb^	3.4±0.6^b^
Test statistics^1^		*F*_2,943_ = 13.75 *P* = 0.006	*F*_2,134_ = 2.34 *P* = 0.147	*P* = 0.017	*P* = 0.670	*F*_2,943_ = 32.52 *P* = 0.0001	*F*_2,943_ = 8.82 *P* = 0.012	*F*_2,943_ = 19.52 *P* = 0.0007	*F*_2,134_ = 8.50 *P* = 0.042
*Larger-scale farms*	N	Lying	Milking	Lying	Milking	Lying	Milking	Lying	Milking
Calm	50	72.7±3.2^a^	79.1±3.8^a^	27.2±6.8^a^	16.8±5.6	46.2±7.0^Aa^	22.6±6.9^a^	1.4±0.4^Aa^	3.3±0.6^a^
Intermediate	50	74.6±4.4^a^	83.2±4.7^ab^	22.2±7.3^a^	12.9±4.5	36.6±10.7^Ab^	19.3±7.4^ab^	2.2±0.8^Ab^	3.6±1.0^ab^
Temperamental	48	81.2±4.5^b^	92.4±4.4^b^	15.2±7.6^b^	12.4±3.9	21.1±3.7^B^	16.7±4.5^b^	2.9±1.1^B^	4.2±1.3^b^
Test statistics^1^		*F*_2,1120_ = 7.34 *P* = 0.016	*F*_2,148_ = 7.25 *P* = 0.026	*P* = 0.026	*P* = 0.425	*F*_2,1120_ = 26.05 *P* = 0.0002	*F*_2,148_ = 7.74 *P* = 0.008	*F*_2,1120_ = 9.30 *P* = 0.008	*F*_2,148_ = 6.92 *P* = 0.023

^1^Statistics are based on the output for the Repeated Measures ANOVA in case of HR, HF and LF/HF (mean ± SD) and on the Friedman rank sum test in case of RMSSD (median ± MAD). MAD = the median of the sorted absolute differences (between the single values and the median of the whole sample). Statistical significances between temperament groups are based on Tukey’s test and on the result of the Neményi *post-hoc* test (^ab^ *P* < 0.05, ^AB^ *P* < 0.01).

### BRH and cardiac autonomic activity

Heart rate and HRV parameters for BRH groups are presented in Table 4. Basal heart rate did not differ between groups either on smaller- or on larger-scale farms. RMSSD was higher in reserved cows than in impulsive ones on farms of both sizes, suggesting a higher parasympathetic dominance of reserved animals. RMSSD of intermediate animals did not differ from that of other groups either on smaller- or on larger-scale farms.

**Table 4:** HRV parameters of dairy cows categorized by behavioural reactivity to humans calculated for lying and milking.

Behavioural reactivity to humans (BRH)		HRV parameter							
		Heart rate (min^–1^)		RMSSD (ms)		HF (n.u.)		LF/HF	
*Smaller-scale farms*	N	Lying	Milking	Lying	Milking	Lying	Milking	Lying	Milking
Impulsive	49	68.2±2.9	77.8±3.5	25.7±8.3^a^	16.8±8.3	33.4±4.4^Aa^	18.9±9.3	2.0±0.3^Aa^	2.6±1.2
Intermediate	46	67.7±3.0	78.0±3.9	32.7±7.6^ab^	15.2±7.9	43.6±5.6^Ab^	22.0±7.9	1.2±0.4^b^	2.2±0.9
Reserved	40	68.5±3.1	77.5±4.1	40.3±5.5^b^	14.7±6.6	54.1±7.2^B^	21.7±8.6	0.9±0.3^Bb^	1.8±0.8
Test statistics^1^		*F*_2,943_ = 1.14 *P* = 0.990	*F*_2,134_ = 1.38 *P* = 0.985	*P* = 0.038	*P* = 0.820	*F*_2,943_ = 22.06 *P* = 0.0007	*F*_2,134_ = 1.80 *P* = 0.715	*P* = 0.008	*P* = 0.238
*Larger-scale farms*	N	Lying	Milking	Lying	Milking	Lying	Milking	Lying	Milking
Impulsive	45	74.0±3.8	81.3±3.9	21.4±4.9^a^	21.4±8.9	30.2±3.9^A^	14.1±5.3	2.2±0.5^a^	3.9±1.3
Intermediate	49	74.3±4.1	82.0±4.2	27.2±6.6^ab^	19.2±7.8	33.6±4.2^ABa^	17.0±6.9	2.0±0.5^a^	4.1±1.4
Reserved	57	76.7±4.2	87.4±4.0	35.4±7.0^b^	18.2±8.7	45.1±6.7^Bb^	16.7±6.6	1.2±0.4^b^	2.3±0.9
Test statistics^1^		*F*_2,1120_ = 1.23 *P* = 0.920	*F*_2,148_ = 2.30 *P* = 0.645	*P* = 0.040	*P* = 0.735	*F*_2,1120_ = 12.65 *P* = 0.005	*F*_2,148_ = 3.37 *P* = 0.830	*P* = 0.035	*P* = 0.125

^1^Statistics are based on the output for the Repeated Measures ANOVA in case of HR and HF (mean ± SD) and on the Friedman rank sum test in case of RMSSD and LF/HF (median ± MAD). MAD = the median of the sorted absolute differences (between the single values and the median of the whole sample). Statistical significances between BRH groups are based on the result of Tukey’s test and on the result of the Neményi post-hoc test (^ab^ *P* < 0.05, ^AB^ *P* < 0.01).

Differences in HRV parameters between intermediate animals and the other behavioural groups were found in HF and LF/HF. In smaller-scale farms, intermediate cows were characterized by higher vagal and lower sympathetic activity than impulsive ones (higher HF and lower LF/HF), but their ANS activity did not differ from that of reserved ones. On larger-scale farms, intermediate animals did not differ in spectral indices from impulsive cows, but the higher HF and lower LF/HF reflected a higher vagal tone compared to reserved cows.

Moderate differences were observed between BRH groups in RMSSD in the case of both farm sizes and in LF/HF on larger-scale farms (*P* < 0.05). Differences between impulsive and reserved cows in basal ANS activity were highly significant on smaller- and larger-scale farms (*P* < 0.01 in both cases), indicating the higher resting vagal (higher HF) and lower sympathetic activity (lower LF/HF) of reserved animals compared to impulsive ones.

There was no difference in heart rate or in HRV parameters between groups during milking, measured either on smaller- or on larger-scale farms.

## Discussion

Experimental studies on the behaviour of animals kept in intensive housing systems describe a cascade of behavioural responses to aversive situations. In this paper, we used two different groupings of dairy cows, one based on the degree of restlessness during an unfamiliar procedure (temperament groups) and one based on behavioural differences in the animal’s reaction to an approaching person (BRH groups), to evaluate individual differences in cardiac autonomic activity. Instead of exposing animals to novel situations, heart rate and ANS-related HRV parameters were recorded in the animals’ familiar environment, during resting in the barn and during milking, the latter in the presence of an unfamiliar person. The small number of studies reported earlier divided individuals into subgroups depending on their behavioural responses to stressors, e.g. high or low avoidance [38], high or low responder [4] and calm or temperamental [3]. In these papers differences between animals have been studied only in situations where animals were exposed to experimentally induced stressors. Therefore, one of the main objectives of our study was to test whether these differences are remarkable under resting conditions as well.

In the present study, cow temperament was related to differences in ANS activity on both smaller-scale and larger-scale farms. According to our hypothesis, the results indicated a higher basal sympathetic and lower vagal activity in temperamental cows than in calm ones. Specifically, we observed higher heart rate, LF/HF and lower RMSSD and HF in temperamental cows than in intermediate or calm animals. Since this is the first paper describing differences in baseline ANS function between bovine animals with different behavioural characteristics, it is difficult to compare our results to earlier findings in this field. In line with our results, higher basal heart rates were found in cows with high behavioural reactivity compared with low responders [4]; however, the cited authors analyzed HRV data obtained while animals were standing in the pre-milking holding pen, which prevented the adequate interpretation of results. According to our earlier studies, heart rate and HRV recorded while cows are waiting to be milked [35] or standing [25,34] differ from resting values, and thus relevant conclusions are difficult to draw from them. In their recent study, Frondelius et al. [25] attempted to describe relationships between baseline HRV parameters and the emotional reactivity of cows; however, they obtained contradictory results as both vagal measures (RMSSD and HF) and the LF parameter (an index of sympathetic activity) correlated positively with a tendency to avoid handling.

In this work, we observed similar differences both in time- (RMSSD) and in frequency-domain HRV (HF, LF/HF) between calm and temperamental groups on exposure to an unfamiliar person during milking as during lying. Temperamental cows from smaller-scale farms had higher heart rates during resting conditions but not during milking than calm ones, whereas temperamental cows from larger-scale farms had higher heart rates in both situations than calm animals. In line with our results, high responder cows had higher heart rates compared to low responders in a rotary milking parlour [4]. Although no experimental data are available on cow’s physiological responsiveness to milking in different sizes of milking parlours, it seems that the observed differences are management related. Seeing that in the investigated smaller-scale farms 2×5-stall herringbone parlours were in operation, while in larger-scale farms 2×2×22-stall parallel parlours, one explanation of our results could be a higher level of social stress on larger-scale farms worked with larger milking parlours and milked 88 cows at the same time. As temperamental cattle are more stress responsive than calmer ones [3] and in a field study we found that crowding in the milking parlour’s holding pen means considerable stress in large milking parlours [35] it is possible that the higher heart rates of temperamental cows were the persistent effect of the pre-milking period. As ANS-related HRV indices mirrored higher stress load in temperamental animals compared to calm cows on both farm sizes, the interpretation of our data may need further investigation.

Several studies have focused on inter-individual differences in behaviours, and some found that coping styles correlate with physiological traits [2,39]. So far, behavioural responses to humans have been studied by means of monitoring only the heart rate in dairy cows [32], but mainly by using tests in environments unfamiliar to the animals [15]. In this study, we evaluated heart rate and ANS-related HRV parameters in cows categorized by BRH when animals were lying without any kind of disturbance and during milking in the presence of an unfamiliar person.

We observed similar differences between groups on smaller- and larger-scale farms. According to our results, BRH is related to differences in basal ANS activity during lying but not during milking. Besides RMSSD, we used the HF component, which is the most informative HRV parameter representing vagal regulatory activity in dairy cattle [32]. As could be expected, reserved cows had higher basal vagal (higher RMSSD and HF) and lower basal sympathetic activity (lower heart rate and LF/HF) than impulsive animals. On the ground of our basic supposal according to BRH may closely related to cattle reactivity, our findings support the results of Engel and Schmale [13] and a review on coping styles in animals [10] emphasizing that proactive coping style is characterized by a low vagal tone while reactive coping style by a strong cardiac parasympathetic activity.

Experiments that investigated differences between individuals in HRV parameters were designed for studying coping styles in response to stressful situations. This could be the reason why the results are inconsistent. For instance, Korte et al. [18] found that reactive laying hens had higher vagal tone represented by RMSSD than did proactive laying hens in response to a restraint test, whereas in proactive pigs higher RMSSD was observed than in reactive ones in response to a novel environment test and novel object test [40]. However, in a more recent study, RMSSD did not differ between proactive and reactive pigs [41].

Numerous studies evaluated cattle’s fear responses to humans from several aspects using different experimental designs [42,43]. According to a recent work, reactivity to humans is mainly influenced by the previous experience of an animal towards humans, and in cattle it can be influenced by fear [44] resulting in higher avoidance [45]. We used the avoidance distance as the main component in our formula used for determination of BRH. In our work, heart rate and ANS-related HRV parameters were calculated for individuals grouped by BRH as a possible component of coping style during resting conditions without human exposure. Based on our findings, it is possible that BRH is rather an individual trait driven by internal regulation than an unambiguous consequence of handling. This is in line with another statement [20], according to which reactivity can be defined as a correlated set of individual behavioural and physiological characteristics.

We found no differences in heart rate and HRV between BRH groups when animals were milked in the presence an unfamiliar person. Studies on heart rate and HRV of farm animals differing in reactivity are still lacking. The small number of works studying ANS characteristics demonstrated neuroendocrine differences in laboratory animals. An earlier study reported on higher sympathetic reactivity (higher plasma noradrenaline levels) and concomitantly lower vagal reactivity (increased heart rate and decreased HRV) of aggressive wild-type rats exposed to social defeat than in less aggressive Wistar rats [17]. Other authors found that proactive coping rodents show a high sympathetic reactivity (high levels of catecholamines) in response to stressful stimulation, whereas behaviourally reactive rodents are characterized by higher vagal reactivity [46].

In our study, the differences observed between BRH groups during lying diminished when a person unfamiliar to the cows was present during milking. The same pattern was observed on farms of both sizes. One of the reasons could be that behavioural characteristics based on fear-tests are not necessary correlated with physiological stress reactions when recorded in the presence of people [47]. Another explanation might be that during milking a cascade of stimuli (physical activity involved in standing [25,34], the presence of milkers, herd mates, and the applied milking technology [35,48] may affect an individual’s physical and emotional state, which is mirrored in cardiac autonomic activity in several ways. It is also debatable whether milking with the presence of an unfamiliar person was an appropriate condition for studying physiological responsiveness using heart rate and HRV parameters. This situation may have even induced a positive emotional state in animals having previous gentle interactions with people [49] whereas for animals with harmful experiences the milking was more aversive [47,50] independently from being impulsive or reserved.

In conclusion, heart rate and HRV seem to be relevant variables to quantify individual differences in behavioural aspects. Our results point out that grouping cows on the basis of BRH made it possible to demonstrate differences between and basal ANS activity of individuals; however, it seems difficult to distinguish between ANS activity of animals with different reactivity to humans in stressful situations. As basal cardiac autonomic activity differs with cow’s temperament and BRH, we recommend to consider these aspects of personality when selecting animals for studies investigating heart rate and HRV parameters for more unbiased results and their more adequate interpretation than before. The analysis of heart rate and HRV may be fundamental for a better understanding of the physiological aspects of behavioural responsiveness in dairy cattle.
